# Mental Health: Pandemics, Epidemics and Tau Protein

**DOI:** 10.2174/17450179-v19-e230510-2022-51

**Published:** 2023-06-21

**Authors:** Ghinwa M. Barakat, Ghaith Assi, Noura B. El Khoury

**Affiliations:** 1 Department of Biological and Chemical Sciences, School of Arts and Sciences, Lebanese International University, Beirut, Lebanon; 2 Department of Neuroscience, Gilbert and Rose-Marie Chagoury School of Medicine, Lebanese American University, Beirut, Lebanon

**Keywords:** Mental health, Depression, Anxiety, PTSD, COVID-19, Pandemics/Epidemics, Tau, Neurotransmitters

## Abstract

**Background:**

It is well established that a wide range of psychological disorders are influenced by the way people live, with lifestyle-related factors playing a substantial role. During the past decade, the effects of major disasters on mental health have drawn a lot of attention.

**Aim:**

In this review, we compare clinical studies reporting a link between COVID-19 and other pandemics and mental health. Importantly, we also shed light on Tau protein and neurotransmitters as neurobiological factors that might explain this link.

**Methods:**

A thorough PubMed search was done to gather and summarize published data on the COVID-19 pandemic’s effect on mental health. Additionally, these studies were compared to previous research published on PubMed, triggering other pandemic and epidemic impacts on mental health.

**Results:**

The COVID-19 epidemic has had the biggest impact on raising awareness about mental health. Moreover, the past century has seen an increase in the frequency of disease outbreaks like MERS-CoV, Ebola, and Influenza, which all had an impact on mental health. However, the exact role of these epidemics on mental health and brain functions is poorly understood.

**Conclusion:**

Future research on the underlying pathways may yield essential information for the treatment and prevention of prospective mental diseases in light of the ongoing decline in mental health during the past 10 years.

## 1 INTRODUCTION

### 1.1 Mental Health: Then and Now

Different systems, cultures, or therapeutic practices have different ideas about what constitutes mental and social health [1]. According to World Health Organization, mental health is a condition of well-being in which a person is aware of their own potential, able to deal with everyday stressors, capable of productive and fruitful employment, and able to give back to their community [2]. According to this concept, having a healthy mental state requires more than just the absence of mental disorders [3]. Hence, modern clinical psychiatry now places a strong emphasis on preventing mental illnesses. The indicated prevention of mental diseases lowers subthreshold symptoms in those who are at risk, which will ultimately result in a reduction in the prevalence and incidence of mental disorders [4]. However, these prevention strategies are insufficient because they typically do not aim to reach the entire population [5]. Moreover, the focus on medical disease has dominated that of clinical psychiatry for the past several decades [6]. For this reason, research efforts in the mental health area have been irregular and inconsistent [7]. Nevertheless, following the rapid increase in mental health issues and especially after COVID-19 pandemic, this topic is witnessing a revival and reconsideration.

### 1.2 Mental Health: The Player-makers

Numerous psychological conditions are known to be caused or affected by how people live, with related lifestyle variables having a significant role in this. For instance, many epidemiological studies have revealed that lifestyle elements, including nutrition, exercise, mental activity, and stress have a big impact on how long people can live with a healthy mentality [8]. These factors can have either a negative or a positive effect.

For instance, Obesity, which has been increasing dramatically over the past 70 years [9], has contributed to an increase in metabolic diseases like type 2 diabetes, which has been linked to psychiatric disorders like depression [10].

The body of studies supporting the improvement and prevention of psychiatric diseases through altering certain lifestyle factors is steadily expanding [11]. For example, smoking has been proven to significantly increase the risk of morbidity and mortality through the emergence of numerous cancers and other illnesses. This, in turn, have a detrimental effect on mental health [12]. A study reported that smokers experienced higher drop-out rates, greater mental discomfort, and worse quality of life from the start to a six-month follow-up period as compared to non-smokers [13].

Another study showed that exercise plays a positive major role on mental health in particular mood states such as anxiety, depression, and stress through different body mechanisms [14]. For example, it was reported that exercise improved mental health and reduced the need of care in schizophrenic patients [15].

Although social connections typically play a positive function on an individual’s mental health, their protectiveness differs between groups and sexes. Men and women seem to receive different kinds of social support from social networks [16]. Yet, given the evolving complexity of human interactions, it is oversimplified to assume that everyone, regardless of age, gender, educational attainment, social status, and cultural background, will benefit from increased social networking, especially online [17].

### 1.3 Mental Health: Disasters Aftermath

The consequences of significant disasters on mental health have received a lot of attention. There is a sizable and quickly expanding body of research on the effects of disasters on affected populations' mental health. PTSD (Post-Traumatic Stress Disorder) is typically reported to be the most prevalent disorder in populations exposed to disasters and has historically been the disorder associated with catastrophes that have been studied the most [18]. Following exposure to trauma, psychiatric comorbidity with PTSD is quite common, especially major depressive disorder and substance abuse [19]. This agrees with most disaster research that has shown major depression as the second most common post-disaster disorder [20]. Moreover, alcohol and drug use have been found to increase following disasters, according to research on the topic [21]. For additional post-disaster psychiatric diseases such as generalized anxiety disorder, panic disorder, and adjustment disorder, relatively fewer studies have provided prevalence data. In these studies, the prevalence rates of these diseases are typically much lower than those for PTSD and major depression [22]. COVID-19 (Corona Virus Disease-19), being a health “disaster” or crisis, would also trigger the curiosity to research its impact on mental health. Not only this, but it would also prompt an interest in relating biological factors to mental health.

#### 1.3.1 Aim

In this review, we discuss the impact of the COVID-19 pandemic compared to other previous pandemics and shed light on the Tau protein and neurotransmitters as neurobiological factors that play a role in mental health.

## 2 METHODOLOGY

A thorough PubMed search was done to gather and summarize published data on the COVID-19 pandemic’s effect on mental health. Additionally, these studies were compared to previous research published on PubMed on other pandemic and epidemic impacts on mental health.

## 3 RESULTS

### 3.1 COVID-19: Quarantine and Mental Health

The contagious illness COVID-19 is brought on by the SARS-CoV-2 virus. The COVID-19 outbreak was accompanied by a drop in positive emotions and life content among the general public, as well as an immediate rise in negative emotions, including anxiety and depression [23]. For instance, according to a study conducted in Wuhan, 20% of Wuhan students who were required to stay at home reported experiencing anxiety and depression [24]. The long-term psychiatric effects of COVID-19 are probably similar to those seen during the SARS (Severe Acute Respiratory System) outbreak (which started in February 2003 and lasted for six months), so that after physical recovery, 50% and 20% of survivors continued to show signs of anxiety and depression, respectively, and people who were given antiviral and corticosteroid medications also showed strong memory problems [25]. Additionally, it appeared that the psychological discomfort in quarantine settings, like social distancing or isolation, was adversely related to the advised health behaviors to reduce the chance of infection [26].

According to a recent analysis of the effects of quarantine and similar preventive measures on mental health, people who have experienced physical isolation are much more likely to experience depression, anxiety disorders, mood disorders, sleep disorders, panic attacks, posttraumatic stress symptoms, stigmatization, low self-esteem, and a lack of self-control [27].

A study conducted by Wang and his colleagues showed that a moderate to severe mental health concern, such as signs of depression, anxiety, insomnia, or acute stress, was reported by 29.2% of the 56,679 participants from 34 Chinese provinces [28].

Another quick examination revealed that widespread posttraumatic stress symptoms, disorientation, and anger might have been brought on by stressors such as extended quarantine, fear of infection, annoyance, boredom, insufficient supplies, poor information, financial loss, and stigma [29].

### 3.2 COVID-19: Previous Diseases and Mental Health

Another issue with regard to global health is how COVID-19 psychologically affects people who test positive [30]. Patients with previous diseases or those with poor access to healthcare are more likely to experience psychological stress during this epidemic [31]. Additionally, prior to the pandemic, some individuals and populations may have experienced a number of mental health issues [32], which could make them more vulnerable to negative mental health outcomes after receiving a COVID-19 diagnosis. There is even more research indicating that COVID-19 patients may experience depression, anxiety disorders, psychological distress, and suicidal behavior [33], which calls for a thorough study of the mental health epidemiology during this pandemic. A study revealed that changes in distress caused by COVID-19 were explained by changes in the severity of the coronavirus symptoms; distress was more prominent in individuals whose symptoms were more severe and took longer to go away [34]. Moreover, according to the research conducted by Xie and his colleagues, those who survive the acute stage of COVID-19 are more likely to experience a variety of incident mental health conditions [35]. Combating mental health issues among COVID-19 survivors needs to be a top concern. Not to mention the increased suicide rates, which must also be taken into account [36]. Suicide rates appear to be linked to the effects of the lockdown-related economic slowdown, such as unemployment and fear of infection, especially in low-income nations [37].

### 3.3 COVID-19: Front Liners and Mental Health

It is also worth mentioning that COVID-19 may also have an impact on the healthcare personnel’s mental health and general wellness, particularly those who serve as frontline providers [38]. An increase in working hours for many healthcare workers that are needed to address the increasing demand for critical care as COVID-19 cases have an impact on the capacities of health systems around the world. These factors were shown to increase the risk of anxiety, sadness, burnout, and insomnia [39]. In an Italian study of oncology specialists, the majority of whom were doctors, 69.2% of the participants reported deteriorating mood, 92% reported concerns about the virus, and 59.5% revealed anxiety over postponing cancer patients' therapies [40]. Moreover, depression symptoms considerably rose during the pandemic compared to pre-pandemic levels, according to a study that looked at doctors in China prior to COVID-19 (2018-2019) and during the pandemic (2019-2020) [41]. Not to mention, in Saudi Arabia, during COVID-19, 56.9% of physicians reported feeling isolated [42].

## 4 OTHER HEALTH CRISIS AND MENTAL HEALTH; PANDEMICS AND EPIDEMICS

Population growth, the world's growing interconnection, microbiological adaptation and change, economic development, changes in land use, and climate change have all contributed to a rise in the frequency of disease outbreaks during the past century. Hence, COVID-19 is not the only pandemic, but it is one of the many that also had a great impact on the population’s mental health. For instance, during the MERS-CoV epidemic, compared to individuals who had not been exposed, nurses who had been exposed to confirmed or suspected MERS cases showed a higher level of stress [43]. In addition, higher degrees of anxiety were reported by non-physicians about the possibility of spreading MERS-CoV to their family [44].

This case was not different during the Ebola epidemic, as healthcare workers (HCWs) in all three of the impacted countries, as well as Ebola survivors in Guinea and Sierra Leone, have all been shown to be in very high levels of distress [45]. Moreover, after a year of the Ebola response, PTSD and anxiety-depression symptoms were widespread, and those who had Ebola-related experiences could require psychosocial care [46].

This is also the case during influenza epidemics, where, in contrast to bipolar disorder, case-control research reported that people who were exposed to an influenza epidemic during the second trimester of fetal development are more likely to acquire unipolar affective disorders, which supports the neuro-developmental concept of the affective disorder [47]. This means that the epidemic had only depressive episodes without any manic ones, which were shown to be linked to neurodevelopment. Yet, compared to employees working in low-risk environments, those in high-risk workplaces were more stressed and exhausted during the H1N1 epidemic [48].

On the other hand, SARS patients typically experience post-traumatic stress disorder (PTSD) while recovering, with one study finding that the lowest blood oxygen saturation (SaO2) level during hospitalization was the most important predictor for intrusion and avoidance symptoms [49]. It is also significant to highlight that, although earlier investigations were not intended to make a psychiatric disease diagnosis, they revealed that 41–65% of SARS survivors had persisting psychological symptoms [50]. The finding that SARS patients who were healthcare workers are at an elevated risk of PTSD (40.7%) is also in line with a prior study's findings [51], as well as the discovery that health professionals who treated SARS patients but were not infected continued to experience significant psychological distress [52] (Table **1**).

## 5 NEUROLOGICAL MECHANISM: TAU HYPERPHOSPHORYLATION

Although a large number of studies have investigated the role of epidemics on mental health, the neurological mechanisms underlying this relationship are still poorly understood. Tau is a microtubule-associated protein (MAP) expressed in neurons. Its function is to regulate the normal operation of the cytoskeletal network in terms of microtubule assembly [53]. Importantly, the most prevalent form of dementia is Alzheimer's Disease (AD), a chronic neurodegenerative condition [54]. Two key characteristics of AD include the extracellular buildup of amyloid-beta (Aβ) peptides as amyloid plaques and the formation of intraneuronal neurofibrillary tangles (NFTs) from hyperphosphorylated or abnormally phosphorylated Tau protein [55]. The presence of NFTs is a characteristic of a group of degenerative diseases known as Tauopathies [56].

In addition to its contribution to AD, Tau was shown to play a role in mental health disorders. For example, in one imaging study, it was reported that depression was twice as likely to be present in participants with elevated Tau. Similarly, participants with increased Tau who were taking antidepressants had a higher likelihood of experiencing depression [57]. Moreover, in another study, increased Tau binding on PET was found to be significantly linked with cognitive impairment in the PTSD (Post Traumatic Stress Disorder) population [58].

Interestingly, postmortem studies also reported a link between Tau hyperphosphorylation and mental health. For example, a recent study reported the presence of hyper-phosphorylated Tau in the postmortem brains of bipolar patients [59] at the Thr231 site, which is known to be important in microtubule assembly and stabilization [60]. Therefore, this will affect major cellular mechanisms such as axonal transport and neuronal differentiation [61]. Moreover, it was reported that patients with schizophrenia had considerably lower blood total Tau and phosphorylated Tau levels in comparison to healthy controls [62]. Further studies are needed in this context, as Tau peripheral levels could be different from those in the brain.

It is important to mention that some studies focused on metals’ role in neurodegeneration. Four metal ions (Al, Zn, Fe, and Cu) may have diverse effects on the development of amyloid aggregates [63]. For instance, amyloid protein precursors are copper-binding proteins [64]. Metal ions may impact the aggregation of amyloid beta (Aβ) monomers from random coil Aβ monomers to beta-sheet monomers, resulting in the production of mature fibrils that eventually settle as senile plaques [65].

## 6 NEUROLOGICAL MECHANISMS: NEUROTRAN-SMITTERS UNBALANCE

The onset and progression of depression and anxiety were tightly correlated with the central nervous system's neurotransmitter alterations [66]. Through meta-analysis, Romeo and his associates demonstrated that patients with major depressive disorder (MDD) have lower GABA expression levels in their brains and peripheral tissue [67]. Moreover, numerous neuropsychiatric conditions were linked to dopamine and its receptor abnormalities, which also contributed to the development of depression [68]. Also, depression and serotonin levels in the brain were proven to be intimately connected [69]. There are reports from a study conducted by Hermann and his team supporting norepinephrine's involvement in dementia due to the loss of noradrenergic neurons in Locus Coeruleus (LC) [70], and it has also been discovered by Mathews and his colleagues that variations in this neurotransmitter's typical function are linked to AD [71]. Moreover, in the CNS, astrocytes are the primary sites of viral infection, according to a new post-mortem analysis, and cells infected with SARS-CoV-2 show noticeable metabolic alterations [72]. However, the restoration of the glial physiological state, whether by the astrocytes or even the microglial cells, may not take place in some individuals. This, in turn, compromises glial function and ultimately leads to homeostatic failure, which underlies a specific set of neuropsychiatric symptoms associated with COVID-19 [73].

## CONCLUSION

In conclusion, several clinical studies have found a link between mental health disorders and pandemics or epidemics, such as COVID-19, ebola, and influenza. Although the link is still poorly understood, studies converge to indicate that Tau hyperphosphorylation and neurotransmitter imbalance might represent possible neurological mechanisms. In light of the continuous decline in mental health over the last decade, future focus on the underlying mechanisms may provide invaluable information for the treatment and prevention of potential mental disorders.

## AUTHORS’ CONTRIBUTIONS

All authors contributed equally to this work. All authors read and approved the final manuscript.

## Figures and Tables

**Table 1:** Epidemics and Pandemics: Studies on their effects in different targeted populations.

**Pandemic (P)/Epidemic(E)**	**Year of Study**	**Type of Mental Disorder**	**Affected People**	** Results **
MERS-CoV (E)	2017	Stress	Nurses	“When compared to individuals who had not been exposed, the nurses that had been exposed to confirmed or suspected MERS cases showed a higher level of stress.”
MERS-CoV (E)	2019	Anxiety	Non-physicians	“Higher degrees of anxiety were reported by non-physicians about the possibility of spreading MERS-CoV to their family.”
Ebola (E)	2017	Distress	Health Care Workers	“Healthcare workers (HCWs) in all three of the impacted countries, as well as Ebola survivors in Guinea and Sierra Leone, have all been shown to be in very high levels of distress.”
Ebola (E)	2018	PTSD Anxiety Depression	Population in affected countries	“After a year of the Ebola response, PTSD and anxiety-depression symptoms were widespread, and those who had Ebola-related experiences may require psychosocial care.”
Influenza (E)	1997	Unipolar affective disorders	Exposed population	“The people who were exposed to an influenza epidemic during the second trimester of their fetal development are more likely to acquire unipolar affective disorders, which supports the neuro-developmental concept of affective disorder.”
Influenza (H1N1) (E)	2012	Stress	High-risk workplaces	“High-risk workplaces had more stressed and exhausted personnel during the H1N1 epidemic.”
SARS (P)	2005	PTSD	SARS patients	“SARS patients typically experienced post-traumatic stress disorder (PTSD) while recovering.”
SARS (P)	2006	Psychological symptoms	SARS survivors	“41–65% of SARS survivors had persisting psychological symptoms.”
SARS (P)	2007	PTSD	SARS patients who were healthcare workers	“SARS patients who were healthcare workers are at an elevated risk of PTSD (40.7%).”
SARS (P)	2006	Distress	Health professionals who treated SARS patients	“Health professionals who treated SARS patients but were not infected continued to experience significant psychological distress”

## Data Availability

The authors confirm that the data supporting the findings of this study are available within the article and its supplementary materials.

## LIST OF ABBREVIATIONS

PTSD: = Post- traumatic Stress Disorder
AD: = Alzheimer's Disease
Aβ: = Amyloid-beta
MDD: = Major Depressive Disorder
NFTs: = Neurofibrillary Tangles

## References

[1] Manwell L.A., Barbic S.P., Roberts K., Durisko Z., Lee C., Ware E., McKenzie K. (2015). What is mental health? Evidence towards a new definition from a mixed methods multidisciplinary international survey.. BMJ Open.

[2] World Health Organization (2004). Prevention of mental disorders: Effective interventions and policy options.. https://apps.who.int/iris/bitstream/handle/10665/43027/924159215X_eng.pdf.

[3] Fusar-Poli P., Salazar de Pablo G., De Micheli A., Nieman D.H., Correll C.U., Kessing L.V., Pfennig A., Bechdolf A., Borgwardt S., Arango C., Van Amelsvoort T. (2020). What is good mental health? A scoping review.. European neuropsychopharmacology.

[4] Shatkin J.P. (2019). Mental health promotion and disease prevention: It’s about time.. J. Am. Acad. Child Adolesc. Psychiatry.

[5] Keyes C.L.M., Dhingra S.S., Simoes E.J. (2010). Change in level of positive mental health as a predictor of future risk of mental illness.. Am. J. Public Health.

[6] Arango C., Díaz-Caneja C.M., McGorry P.D., Rapoport J., Sommer I.E., Vorstman J.A., McDaid D., Marín O., Serrano-Drozdowskyj E., Freedman R., Carpenter W. (2018). Preventive strategies for mental health.. Lancet Psychiatry.

[7] Snodgrass J.G., Lacy M.G., Upadhyay C. (2017). Developing culturally sensitive affect scales for global mental health research and practice: Emotional balance, not named syndromes, in Indian Adivasi subjective well-being.. Soc. Sci. Med..

[8] Li Y., Pan A., Wang D.D., Liu X., Dhana K., Franco O.H., Kaptoge S., Di Angelantonio E., Stampfer M., Willett W.C., Hu F.B. (2018). Impact of healthy lifestyle factors on life expectancies in the US population.. Circulation.

[9] Caballero B. (2007). The global epidemic of obesity: An overview.. Epidemiol. Rev..

[10] Stuart M.J., Baune B.T. (2012). Depression and type 2 diabetes: Inflammatory mechanisms of a psychoneuroendocrine co-morbidity.. Neurosci. Biobehav. Rev..

[11] Cotman C.W., Berchtold N.C., Christie L.A. (2007). Exercise builds brain health: Key roles of growth factor cascades and inflammation.. Trends Neurosci..

[12] Aras Y.G., Tunç A., Güngen B.D., Güngen A.C., Aydemir Y., Demiyürek B.E. (2017). The effects of depression, anxiety and sleep disturbances on cognitive impairment in patients with chronic obstructive pulmonary disease.. Cogn. Neurodynamics.

[13] Lien L., Bolstad I., Bramness J.G. (2021). Smoking among inpatients in treatment for substance use disorders: Prevalence and effect on mental health and quality of life.. BMC Psychiatry.

[14] Mikkelsen K., Stojanovska L., Polenakovic M., Bosevski M., Apostolopoulos V. (2017). Exercise and mental health.. Maturitas.

[15] Scheewe T.W., Backx F.J.G., Takken T., Jörg F., van Strater A.C.P., Kroes A.G., Kahn R.S., Cahn W. (2013). Exercise therapy improves mental and physical health in schizophrenia: A randomised controlled trial.. Acta Psychiatr. Scand..

[16] Kawachi I., Berkman L.F. (2001). Social ties and mental health.. J. Urban Health.

[17] Pantic I. (2014). Online social networking and mental health.. Cyberpsychol. Behav. Soc. Netw..

[18] North C.S., Hong B.A., Pfefferbaum B. (2008). P-FLASH: Development of an empirically-based post-9/11 disaster mental health training program.. Mo. Med..

[19] Foa E.B., Stein D.J., McFarlane A.C. (2006). Symptomatology and psychopathology of mental health problems after disaster.. J. Clin. Psychiatry.

[20] Norris F.H., Friedman M.J., Watson P.J., Byrne C.M., Diaz E., Kaniasty K. (2002). 60,000 disaster victims speak: Part I. An empirical review of the empirical literature, 1981-2001.. Psychiatry.

[21] Boscarino J.A., Adams R.E., Galea S. (2006). Alcohol use in New York after the terrorist attacks: A study of the effects of psychological trauma on drinking behavior.. Addict. Behav..

[22] North C.S., Oliver J., Pandya A. (2012). Examining a comprehensive model of disaster-related posttraumatic stress disorder in systematically studied survivors of 10 disasters.. Am. J. Public Health.

[23] Li S., Wang Y., Xue J., Zhao N., Zhu T. (2020). The Impact of COVID-19 epidemic declaration on psychological consequences: A study on active weibo users.. Int. J. Environ. Res. Public Health.

[24] Xie X., Xue Q., Zhou Y., Zhu K., Liu Q., Zhang J., Song R. (2020). Mental health status among children in home confinement during the coronavirus disease 2019 outbreak in Hubei Province, China.. JAMA Pediatr..

[25] Tsang H.W., Scudds R.J., Chan E.Y. (2004). Psychosocial impact of SARS.. Emerg. Infect. Dis..

[26] Ben-Ezra M., Sun S., Hou W.K., Goodwin R. (2020). The association of being in quarantine and related COVID-19 recommended and non-recommended behaviors with psychological distress in Chinese population.. J. Affect. Disord..

[27] Hossain M.M., Sultana A., Purohit N. (2020). Mental health outcomes of quarantine and isolation for infection prevention: A systematic umbrella review of the global evidence.. Epidemiol. Health.

[28] Wang Y., Shi L., Que J., Lu Q., Liu L., Lu Z., Xu Y., Liu J., Sun Y., Meng S., Yuan K., Ran M., Lu L., Bao Y., Shi J. (2021). The impact of quarantine on mental health status among general population in China during the COVID-19 pandemic.. Mol. Psychiatry.

[29] Brooks S.K., Webster R.K., Smith L.E., Woodland L., Wessely S., Greenberg N., Rubin G.J. (2020). The psychological impact of quarantine and how to reduce it: Rapid review of the evidence.. Lancet.

[30] Zhou S.J., Zhang L.G., Wang L.L., Guo Z.C., Wang J.Q., Chen J.C., Liu M., Chen X., Chen J.X. (2020). Prevalence and socio-demographic correlates of psychological health problems in Chinese adolescents during the outbreak of COVID-19.. Eur. Child Adolesc. Psychiatry.

[31] Kavoor A.R. (2020). COVID-19 in people with mental illness: Challenges and vulnerabilities.. Asian J. Psychiatr..

[32] Hossain M.M., Khan N., Sultana A., Ma P., McKyer E.L.J., Ahmed H.U., Purohit N. (2020). Prevalence of comorbid psychiatric disorders among people with autism spectrum disorder: An umbrella review of systematic reviews and meta-analyses.. Psychiatry Res..

[33] Rogers J.P., Chesney E., Oliver D., Pollak T.A., McGuire P., Fusar-Poli P., Zandi M.S., Lewis G., David A.S. (2020). Psychiatric and neuropsychiatric presentations associated with severe coronavirus infections: a systematic review and meta-analysis with comparison to the COVID-19 pandemic.. Lancet Psychiatry.

[34] Daly M., Robinson E. (2021). Acute and longer-term psychological distress associated with testing positive for COVID-19: longitudinal evidence from a population-based study of US adults.. Psychol. Med..

[35] Xie Y., Xu E., Al-Aly Z. (2022). Risks of mental health outcomes in people with COVID-19: Cohort study.. BMJ.

[36] Lennon J.C. (2020). What lies ahead: Elevated concerns for the ongoing suicide pandemic.. Psychol. Trauma.

[37] Mamun M.A., Ullah I. (2020). COVID-19 suicides in Pakistan, dying off not COVID-19 fear but poverty? – The forthcoming economic challenges for a developing country.. Brain Behav. Immun..

[38] Li G., Miao J., Wang H., Xu S., Sun W., Fan Y., Zhang C., Zhu S., Zhu Z., Wang W. (2020). Psychological impact on women health workers involved in COVID-19 outbreak in Wuhan: a cross-sectional study.. J. Neurol. Neurosurg. Psychiatry.

[39] Pappa S., Ntella V., Giannakas T., Giannakoulis V.G., Papoutsi E., Katsaounou P. (2020). Prevalence of depression, anxiety, and insomnia among healthcare workers during the COVID-19 pandemic: A systematic review and meta-analysis.. Brain Behav. Immun..

[40] Ballatore Z., Bastianelli L., Merloni F., Ranallo N., Cantini L., Marcantognini G., Berardi R. (2020). Scientia Potentia Est: How the Italian world of oncology changes in the COVID-19 pandemic.. JCO Glob. Oncol..

[41] Li W., Frank E., Zhao Z., Chen L., Wang Z., Burmeister M., Sen S. (2020). Mental Health of Young Physicians in China during the novel coronavirus disease 2019 outbreak.. JAMA Netw. Open.

[42] Al Sulais E., Mosli M., AlAmeel T. (2020). The psychological impact of COVID-19 pandemic on physicians in Saudi Arabia: A cross-sectional study.. Saudi journal of gastroenterology.

[43] Oh N., Hong N., Ryu D.H., Bae S.G., Kam S., Kim K.Y. (2017). Exploring nursing intention, stress, and professionalism in response to infectious disease emergencies: The experience of local public hospital nurses during the 2015 MERS outbreak in South Korea.. Asian Nurs. Res..

[44] Alsubaie S., Hani Temsah M., Al-Eyadhy A.A., Gossady I., Hasan G.M., Al-rabiaah A., Jamal A.A., Alhaboob A.A.N., Alsohime F., Somily A.M. (2019). Middle east respiratory syndrome coronavirus epidemic impact on healthcare workers’ risk perceptions, work and personal lives.. J. Infect. Dev. Ctries..

[45] Ji D., Ji Y.J., Duan X.Z., Li W.G., Sun Z.Q., Song X.A., Meng Y.H., Tang H.M., Chu F., Niu X.X., Chen G.F., Li J., Duan H.J. (2017). Prevalence of psychological symptoms among Ebola survivors and healthcare workers during the 2014-2015 Ebola outbreak in Sierra Leone: A cross-sectional study.. Oncotarget.

[46] Jalloh M.F., Li W., Bunnell R.E., Ethier K.A., O’Leary A., Hageman K.M., Sengeh P., Jalloh M.B., Morgan O., Hersey S., Marston B.J., Dafae F., Redd J.T. (2018). Impact of Ebola experiences and risk perceptions on mental health in Sierra Leone, July 2015.. BMJ Glob. Health.

[47] Machón R.A., Mednick S.A., Huttunen M.O. (1997). Adult major affective disorder after prenatal exposure to an influenza epidemic.. Arch. Gen. Psychiatry.

[48] Matsuishi K., Kawazoe A., Imai H., Ito A., Mouri K., Kitamura N., Miyake K., Mino K., Isobe M., Takamiya S., Hitokoto H., Mita T. (2012). Psychological impact of the pandemic (H1N1) 2009 on general hospital workers in Kobe.. Psychiatry Clin. Neurosci..

[49] Wu K.K., Chan S.K., Ma T.M. (2005). Posttraumatic stress after SARS.. Emerg. Infect. Dis..

[50] Kwek S.K., Chew W.M., Ong K.C., Ng A.W.K., Lee L.S.U., Kaw G., Leow M.K.S. (2006). Quality of life and psychological status in survivors of severe acute respiratory syndrome at 3 months postdischarge.. J. Psychosom. Res..

[51] Lee A.M., Wong J.G.W.S., McAlonan G.M., Cheung V., Cheung C., Sham P.C., Chu C.M., Wong P.C., Tsang K.W.T., Chua S.E. (2007). Stress and psychological distress among SARS survivors 1 year after the outbreak.. Can. J. Psychiatry.

[52] Maunder R., Lancee W., Balderson K., Bennett J., Borgundvaag B., Evans S., Fernandes C., Goldbloom D., Gupta M., Hunter J., McGillis Hall L., Nagle L., Pain C., Peczeniuk S., Raymond G., Read N., Rourke S., Steinberg R., Stewart T., VanDeVelde-Coke S., Veldhorst G., Wasylenki D. (2006). Long-term psychological and occupational effects of providing hospital healthcare during SARS outbreak.. Emerg. Infect. Dis..

[53] Binder L.I., Frankfurter A., Rebhun L.I. (1985). The distribution of tau in the mammalian central nervous system.. J. Cell Biol..

[54] Laurent C., Buée L., Blum D. (2018). Tau and neuroinflammation: What impact for Alzheimer’s disease and tauopathies?. Biomed. J..

[55] Choi H., Kim H.J., Yang J., Chae S., Lee W., Chung S., Kim J., Choi H., Song H., Lee C.K., Jun J.H., Lee Y.J., Lee K., Kim S., Sim H., Choi Y.I., Ryu K.H., Park J.C., Lee D., Han S.H., Hwang D., Kyung J., Mook-Jung I. (2020). Acetylation changes tau interactome to degrade tau in Alzheimer’s disease animal and organoid models.. Aging Cell.

[56] Wolfe M.S. (2012). The role of tau in neurodegenerative diseases and its potential as a therapeutic target.. Scientifica.

[57] Babulal G.M., Roe C.M., Stout S.H., Rajasekar G., Wisch J.K., Benzinger T.L.S., Morris J.C., Ances B.M. (2020). Depression is associated with tau and not amyloid positron emission tomography in cognitively normal adults.. J. Alzheimers Dis..

[58] Mohamed A.Z., Cumming P., Götz J., Nasrallah F., Department of Defense Alzheimer’s Disease Neuroimaging Initiative (2019). Tauopathy in veterans with long-term posttraumatic stress disorder and traumatic brain injury.. Eur. J. Nucl. Med. Mol. Imaging.

[59] Naserkhaki R., Zamanzadeh S., Baharvand H., Nabavi S.M., Pakdaman H., Shahbazi S., Vosough M., Ghaedi G., Barzegar A., Mirtorabi D., Hedayatshodeh M., Ehsani E., Falahati M., Hajipour M.J., Shahpasand K. (2019). *cis* pT231-Tau drives neurodegeneration in bipolar disorder.. ACS Chem. Neurosci..

[60] Cho J.H., Johnson G.V.W. (2004). Primed phosphorylation of tau at Thr231 by glycogen synthase kinase 3β (GSK3β) plays a critical role in regulating tau’s ability to bind and stabilize microtubules.. J. Neurochem..

[61] Augustinack J.C., Schneider A., Mandelkow E.M., Hyman B.T. (2002). Specific tau phosphorylation sites correlate with severity of neuronal cytopathology in Alzheimer’s disease.. Acta Neuropathol..

[62] Demirel Ö.F., Cetin I., Turan Ş., Yıldız N., Sağlam T., Duran A. (2017). Total Tau and phosphorylated Tau protein serum levels in patients with schizophrenia compared with controls.. Psychiatr. Q..

[63] Fernandez L.L., Carmona M., Portero-Otin M., Naudi A., Pamplona R., Schröder N., Ferrer I. (2010). Effects of increased iron intake during the neonatal period on the brain of adult AbetaPP/PS1 transgenic mice.. J. Alzheimers Dis..

[64] Brown W.R., Thore C.R. (2011). Review: Cerebral microvascular pathology in ageing and neurodegeneration.. Neuropathol. Appl. Neurobiol..

[65] Fanni D., Gerosa C., Rais M., Ravarino A., Van Eyken P., Fanos V., Faa G. (2018). The role of neuropathological markers in the interpretation of neuropsychiatric disorders: Focus on fetal and perinatal programming.. Neurosci. Lett..

[66] Wirbisky S.E., Weber G.J., Lee J.W., Cannon J.R., Freeman J.L. (2014). Novel dose-dependent alterations in excitatory GABA during embryonic development associated with lead (Pb) neurotoxicity.. Toxicol. Lett..

[67] Romeo B., Choucha W., Fossati P., Rotge J.Y. (2015). Meta-analysis of short- and mid-term efficacy of ketamine in unipolar and bipolar depression.. Psychiatry Res..

[68] Jonas R.K., Montojo C.A., Bearden C.E. (2014). The 22q11.2 deletion syndrome as a window into complex neuropsychiatric disorders over the lifespan.. Biol. Psychiatry.

[69] Sharon G., Garg N., Debelius J., Knight R., Dorrestein P.C., Mazmanian S.K. (2014). Specialized metabolites from the microbiome in health and disease.. Cell Metab..

[70] Herrmann N., Lanctôt K.L., Khan L.R. (2004). The role of norepinephrine in the behavioral and psychological symptoms of dementia.. J. Neuropsychiatry Clin. Neurosci..

[71] Matthews K.L., Chen C.P.L.H., Esiri M.M., Keene J., Minger S.L., Francis P.T. (2002). Noradrenergic changes, aggressive behavior, and cognition in patients with dementia.. Biol. Psychiatry.

[72] Crunfli F., Carregari V.C., Veras F.P., Silva L.S., Nogueira M.H., Antunes A.S.L.M., Vendramini P.H., Valença A.G.F., Brandão-Teles C., Zuccoli G.S., Reis-de-Oliveira G., Silva-Costa L.C., Saia-Cereda V.M., Smith B.J., Codo A.C., de Souza G.F., Muraro S.P., Parise P.L., Toledo-Teixeira D.A., Santos de Castro Í.M., Melo B.M., Almeida G.M., Firmino E.M.S., Paiva I.M., Silva B.M.S., Guimarães R.M., Mendes N.D., Ludwig R.L., Ruiz G.P., Knittel T.L., Davanzo G.G., Gerhardt J.A., Rodrigues P.B., Forato J., Amorim M.R., Brunetti N.S., Martini M.C., Benatti M.N., Batah S.S., Siyuan L., João R.B., Aventurato Í.K., Rabelo de Brito M., Mendes M.J., da Costa B.A., Alvim M.K.M., da Silva Júnior J.R., Damião L.L., de Sousa I.M.P., da Rocha E.D., Gonçalves S.M., Lopes da Silva L.H., Bettini V., Campos B.M., Ludwig G., Tavares L.A., Pontelli M.C., Viana R.M.M., Martins R.B., Vieira A.S., Alves-Filho J.C., Arruda E., Podolsky-Gondim G.G., Santos M.V., Neder L., Damasio A., Rehen S., Vinolo M.A.R., Munhoz C.D., Louzada-Junior P., Oliveira R.D., Cunha F.Q., Nakaya H.I., Mauad T., Duarte-Neto A.N., Ferraz da Silva L.F., Dolhnikoff M., Saldiva P.H.N., Farias A.S., Cendes F., Moraes-Vieira P.M.M., Fabro A.T., Sebollela A., Proença-Modena J.L., Yasuda C.L., Mori M.A., Cunha T.M., Martins-de-Souza D. (2022). Morphological, cellular, and molecular basis of brain infection in COVID-19 patients.. Proc. Natl. Acad. Sci. USA.

[73] Steardo L., Scuderi C. (2022). Astrocytes and the psychiatric Sequelae of COVID-19: What we learned from the pandemic.. Neurochem. Res..

